# Increase phosphorus availability from the use of alfalfa (*Medicago sativa* L) green manure in rice (*Oryza sativa* L.) agroecosystem

**DOI:** 10.1038/srep36981

**Published:** 2016-11-11

**Authors:** Xiaoye Gao, Dongyan Shi, Aimin Lv, Shengyin Wang, Shili Yuan, Peng Zhou, Yuan An

**Affiliations:** 1 School of Agriculture and Biology, Shanghai Jiao Tong University, Shanghai 200240, P. R. China; 2 Research Center for Karst Wetland Ecology, Guizhou Minzu University, Guiyang 550025, P. R. China; 3 School of Biology Science, Heze University, Heze 274015, P. R. China; 4 Key Laboratory of Urban Agriculture (South), Ministry of Agriculture, Shanghai, P. R. China

## Abstract

Alfalfa is a good green manure source, but its effect on rice growth has not been fully elucidated. Two green manure species, alfalfa and broad bean (*Vicia faba* L.), and two N fertilizer levels, alone or combination, were applied to a rice field. The results indicated that alfalfa had more pronounced effects on increasing soil labile phosphorus (P) fractions (including NaHCO_3_-Pi, NaOH-Pi), P uptake and soil enzyme activities (dehydrogenase, urease, acid phosphatase and β-glucosidase) than broad bean and N fertilizer. The transformation of NaHCO_3_-Po to labile P regulated by alfalfa played a significant direct and indirect effect on grain yield. Although a much lower N input from alfalfa addition, a similar grain yield with N fertilizer treatment was achieved, and the integration of alfalfa with N fertilizer produced the highest grain yield and P availability, which was associated with the highest urease, acid phosphatase and β-glucosidase activity in soil. These results indicate that alfalfa green manure had a great ability of increasing grain yield through enhancing P availability in rice paddy, which could give us a way to reduce N fertilizer application by enhancing P availability.

Phosphorus (P) is one of the major nutrients needed to sustain plant growth, and was an indispensable nutrient in the progress of plant growth and development. However, P anions are often immobilized in soil through sorption and precipitation with other cations^1^, resulting the limited P availability to plants. Thus, P deficiency has been a key growth limiting factor for plants, which prevents high crop yields without external P inputs^2^. It has been estimated that approximately 60% of the P in agriculture systems rely on the P fertilizer derived from non-renewable phosphate rock, with the remainder coming from recycled P in organic residues such as manure, crop residues and human excreta^2^,^3^. Increasing the proportion of organic P in the total P application would be an effective way to reduce P fertilizer application. According to data from the US Geological Survey, the world’s remaining phosphate rock was estimated at 62 Gt, and the demand for P fertilizer is predicted to grow by 2.5–3.0% per year. If this rate continues, the world’s P reserves should last for approximately 125 years^4^ and will be depleted in the 21^st^ century^5^,^6^. Therefore, there are serious challenges involved in sustaining the supply of affordable P required for farmers to increase food production to feed an increasing global population. Furthermore, the use efficiency of P fertilizer is as low as 8–33%^7^, which means that a large part of the P fertilizer is immobilized in soil through sorption with other cations^8^, in which a part of the immobilized P will be loss into river and leaks with surface runoff. The net loss of P from the world’s cropland is estimated at approximately 10.5 tons each year, nearly one half of the P extracted yearly^3^, which has led to widespread river eutrophication^9^,^10^. Therefore, P is both a critical and polluting element and must be used more efficiently and sustainably in the future to help safeguard food, energy and water security^11^,^12^,^13^.

Most of the phosphorus applied to agro-ecosystems is often immobilized in soil through sorption and precipitation with other cations^1^. The immobilized P exists in a multitude of chemical forms and pools that become available to plants on different time scales. In normal condition, the P exists in soil in the two forms: inorganic P (Pi) and organic P (Po). The most plausible form of P supplied to plants is from Olsen-P and Pi pools and through mineralization of Po^8^. The organic P mineralization is a biochemical process related to soil microoganisms. Soil enzymes are mainly derived from soil microoganisms, and take part in regulating the biochemical process^14^. Soil microorganisms can increase the utilization of organic P via production of extracellular enzymes to prevent P limitation in highly weathered or strongly acidic soils, and available P pools strongly correlate with microbial composition and soil enzyme production^15^. Phosphatases have been reported to be a major enzyme in mediating the availability of P to plants^16^,^17^,^18^. Soil enzyme production strongly affects biochemical processes involving the decomposition of organic residues and nutrient cycling in soils^19^, and can indicate the potential of soil to support the biochemical processes^14^.

The organic phosphorus is not a significant contributor to soil available P when available inorganic phosphorus is high, however, when soil Pi is low, the Po pools (including NaHCO_3_-Po and NaOH-Po fractions) begin to contribute to available P to supply plant growth^20^. Therefore, organic P is an important source of available P, but it does not appear to affect P availability significantly in high-P mineral soils^20^. The plant-available or labile P fraction consistently makes up <14% of the total P across all soil orders^21^. P deficiency has been identified as a major factor limiting rice yields in many soils around the world^22^. The application of P fertilizer is a key technique for successful rice cultivation. However, the use efficiency of P fertilizer is very low^22^. Increasing P resource efficiency is very important to maintain sustainable agriculture and a sustainable P supply for crop cultivation.

Green manures represent a promising approach to maintaining sustainable nutrients for crop growth^23^. Most of the P in plants has been detected as orthophosphate, which is generally the available form for plant uptake^24^. The P in green manure can potentially be delivered to the soil in a form that is readily available to plants and soil microorganisms^25^, and the soil P adsorption can also be decreased by green manure, as a result to increase available P^26^. Therefore, green manure plays an important function in P supply and P release from mineral-associated P by decreasing the soil P sorption strength on mineral soil particles^27^. Leguminous plants, including broad bean, Chinese milk vetch (*Astragalus sinicus* L.), clover (*Trifolium repens* L.), and vetch (*Vicia sativa* L.) are widely used as green manure in crop rotation systems worldwide^23^,^28^,^29^. Alfalfa is an important perennial legume that is used as forage crop worldwide and it is also a very good green manure resource, with a high P concentration (0.30–0.42% of dry weight) that far exceeds the amount of P in most plants, which ranges from 0.05% to 0.30%^30^. However, limited information is available about the effect of alfalfa green manure on crop culture systems. Cavigelli and Then^31^ reported that alfalfa had the most pronounced effects on sorghum biomass and P uptake increases among four tested green manure plants. In our previous study, we found that alfalfa green manure could increase P accumulation in rice plant, as well as increase grain yields^32^. The mechanism of alfalfa green manure increasing grain yield may be correlated with its improving soil P availability and alleviating P deficiency in rice paddy field. Therefore, in this study, we used the shoots of alfalfa as green manure on a rice field, and compared the alfalfa green manure with broad bean green manure (a local and traditional cultivated green manure crop) and traditional N fertilizer application, alone or combination, in southeast China. The objectives of this study were to determine (1) whether alfalfa green manure increasing grain yield is greatly attributed to its improving soil P availability and alleviating P deficiency in rice paddy field; (2) whether alfalfa has greater potential as a green manure to increase soil P availability than the traditional green manure of broad bean and N fertilizer.

## Results

### Yields of grain and straw

**Figure 1:**
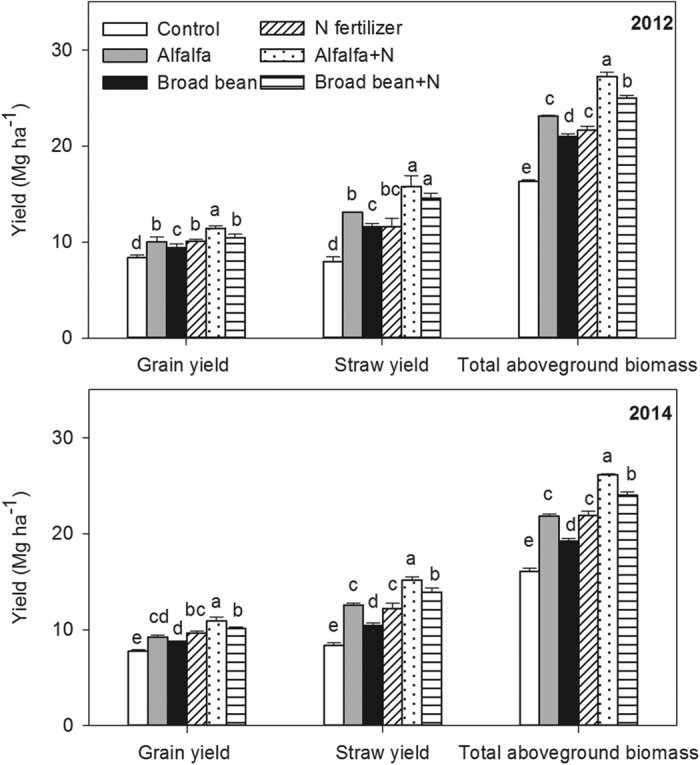
Yields of grain and straw under the following treatments: control (no application), alfalfa (3000 kg DM ha^–1^), broad bean (3000 kg DM ha^–1^), N fertilizer (200 kg N ha^−1^), combined treatment of alfalfa (3000 kg DM ha^−1^) and N fertilizer (200 kg N ha^−1^) (alfalfa + N), and combined treatment of broad bean (3000 kg DM ha^–1^) and N fertilizer (200 kg N ha^−1^) (broad bean + N) in 2012 and 2014. Total aboveground biomass including grain and straw yields. In the same year, the bars with different letters are significant (*P* < 0.05). Data are means ± standard errors.

The alfalfa green manure treatment produced higher grain yield compared to the broad bean green manure treatment (Table 1). The grain yields were 6.32% and 4.86% higher in the alfalfa treatment than the broad bean treatment in 2012 and 2014, respectively (Fig. 1). Compared with the control treatment (no application of green manure and N fertilizer), two green manure treatments and N fertilizer treatment alone significantly increased the yields of grain and straw in 2012 and 2014 (*P* < 0.05). No significant differences (*P* > 0.05) in the yields of grain and straw were found between N fertilizer and alfalfa treatments (Fig. 1). The interaction between N fertilizer and green manure on grain yield was significant (*P* < 0.05) (Table 1). The combined treatment of alfalfa and N fertilizer (alfalfa + N) significantly increased the grain yields by 13.38% and 12.90% in 2012 and 2014, respectively. The grain yields were significantly higher in alfalfa + N treatment than that broad bean + N treatment (*P* < 0.05).

### N concentration in leaves

**Figure 2:**
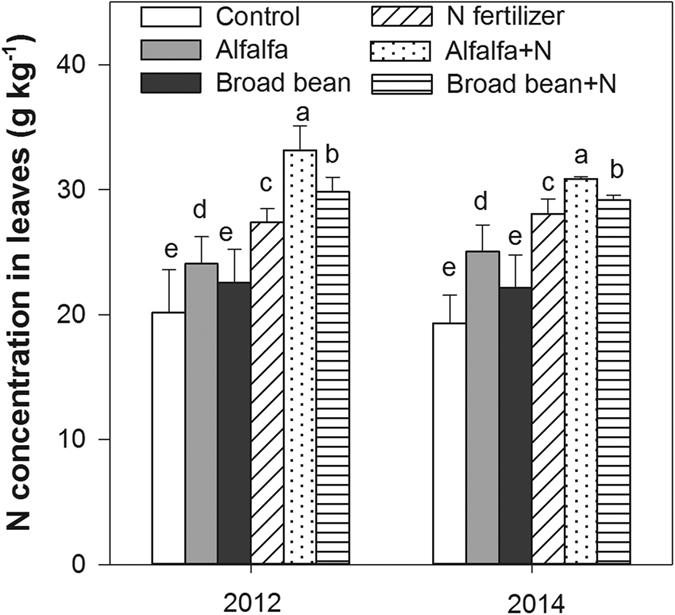
Average N concentrations in leaves during the rice tillering, panicle, and filling stages under the following treatments: control (no application), alfalfa (3000 kg DM ha^–1^), broad bean (3000 kg DM ha–1), N fertilizer (200 kg N ha^−1^), combined treatment of alfalfa (3000 kg DM ha–1) and N fertilizer (200 kg N ha^−1^) (alfalfa + N), and combined treatment of broad bean (3000 kg DM ha–1) and N fertilizer (200 kg N ha^−1^) (broad bean + N) in 2012 and 2014. In the same year, the bars with different letters are significant (*P* < 0.05). Data are means ± standard errors.

Compared with the control treatment, the alfalfa green manure and N fertilizer treatments significantly increased leaf N concentrations in 2012 and 2014, while the N fertilizer treatment increased leaf N concentrations by 13.79% and 12.10% in comparison with the alfalfa treatment, respectively. The leaf N concentrations were significantly higher in the combined treatments of green manure and N fertilizer than green manure treatment alone, with the highest leaf N concentration in alfalfa + N treatment (Fig. 2).

### N concentration and accumulation in grain and aboveground biomass

**Figure 3:**
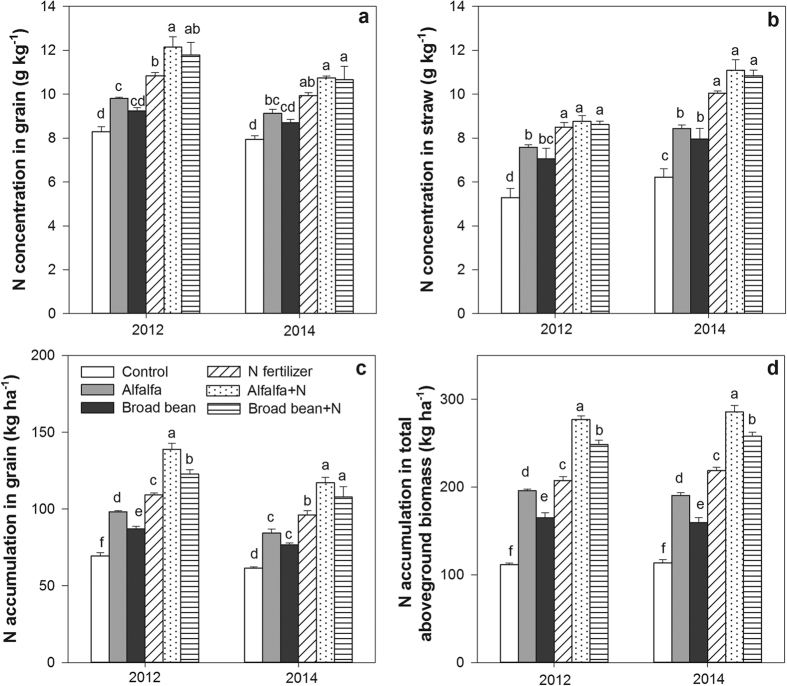
N concentrations in grain and straw and N accumulations in grain and aboveground biomass under the following treatments: control (no application), alfalfa (3000 kg DM ha^–1^), broad bean (3000 kg DM ha^–1^), N fertilizer (200 kg N ha^−1^), combined treatment of alfalfa (3000 kg DM ha^–1^) and N fertilizer (200 kg N ha^−1^) (alfalfa + N), and combined treatment of broad bean (3000 kg DM ha^–1^) and N fertilizer (200 kg N ha^−1^) (broad bean + N) in 2012 and 2014. Total aboveground biomass including grain and straw yields. In the same year, the bars with different letters are significant (*P* < 0.05). Data are means ± standard errors.

The effect of green manures on increasing N concentrations in grain and straw were significantly lower than N fertilizer treatment (*P* < 0.05) (Fig. 3a,b). The treatments of alfalfa and N fertilizer alone significantly increased N concentrations in grain and straw compared to control in 2012 and 2014, while the grain N concentrations were higher in the N fertilizer treatment than alfalfa treatment.

Green manure treatments significantly increased N accumulations in grain and aboveground biomass compared with the control in 2012 and 2014, with the most pronounced effect in the alfalfa treatments (*P* < 0.05) (Fig. 3c,d). But the increased N accumulations in grain and aboveground biomass in alfalfa treatment were lower than N fertilizer treatment (*P* < 0.05). The N accumulations in grain and aboveground biomass were significantly higher in the combined treatments of green manure and N fertilizer than N fertilizer treatment (*P* < 0.05), and the alfalfa + N treatment had the highest N accumulations among all the treatments.

### P concentrations and accumulations in leaves, grain and total aboveground biomass

**Figure 4:**
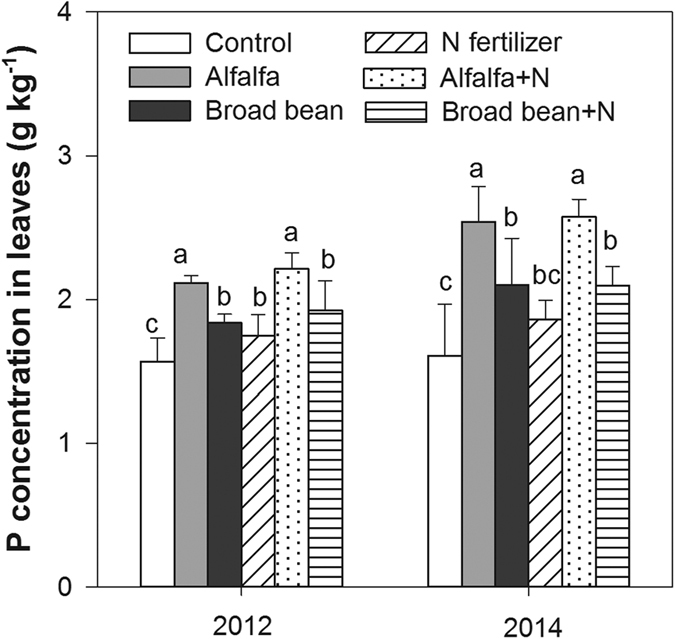
Average P concentrations in leaves during the rice tillering, panicle, and filling stages under the following treatments: control (no application), alfalfa (3000 kg DM ha^−1^), broad bean (3000 kg DM ha–1), N fertilizer (200 kg N ha^−1^), combined treatment of alfalfa (3000 kg DM ha^−1^) and N fertilizer (200 kg N ha^−1^) (alfalfa + N), and combined treatment of broad bean (3000 kg DM ha^−1^) and N fertilizer (200 kg N ha^−1^) (broad bean + N) in 2012 and 2014. In the same year, the bars with different letters are significant (*P* < 0.05). Data are means ± standard errors.

The green manure application had significant effects on P concentrations in leaves and grain, as well as P accumulation in total aboveground biomass (*P* < 0.001) (Table 1). The leaf P concentrations in the alfalfa additions with or without N fertilizer application were significantly higher than all the other treatments, and broad bean additions with or without N fertilizer application significantly increased the leaf P concentrations in comparison with the control treatment (*P* < 0.05) (Fig. 4).

**Figure 5:**
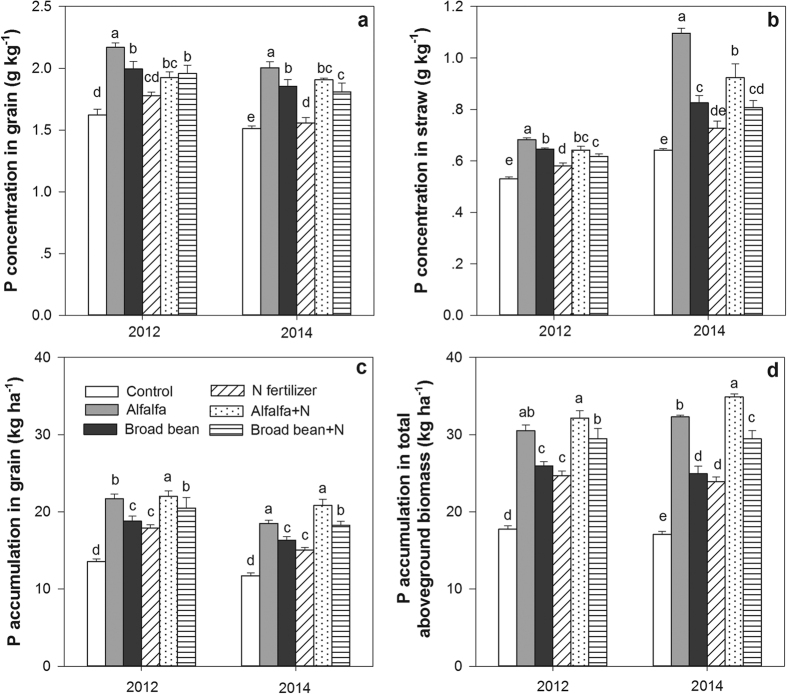
P concentrations in grain and straw and N accumulations in grain and aboveground biomass under the following treatments: control (no application), alfalfa (3000 kg DM ha^–1^), broad bean (3000 kg DM ha^–1^), N fertilizer (200 kg N ha^–1^), combined treatment of alfalfa (3000 kg DM ha^–1^) and N fertilizer (200 kg N ha^–1^) (alfalfa + N), and combined treatment of broad bean (3000 kg DM ha^–1^) and N fertilizer (200 kg N ha^–1^) (broad bean + N) in 2012 and 2014. Total aboveground biomass including grain and straw yields. In the same year, the bars with different letters are significant (*P* < 0.05). Data are means ± standard errors.

Compared to the control and N fertilizer treatments, alfalfa treatment with or without N fertilizer significantly increased the P concentrations in grain and straw, as well as the P accumulations in grain and aboveground biomass in 2012 and 2014 (*P* < 0.05) (Fig. 5), whereas only the P concentrations in grain in 2012 and 2014 and straw in 2012 were significantly higher in the broad bean treatment with or without N fertilizer compared to the control and N fertilizer treatments. The P accumulations in grain and aboveground biomass were significantly higher in the combined treatment of broad bean and N fertilizer compared to the broad bean and control treatments in 2012 and 2014.

The differences in P concentrations in grain were not significant between the N fertilizer and control treatments, but the N fertilizer treatment significantly increased the P accumulations in grain and aboveground biomass. The alfalfa treatments with or without N fertilizer application had the most significant effect on increasing the P concentrations and accumulations among all the treatments (Fig. 5).

### Soil P fractions

Green manure application had significant effect on soil available P content (*P* < 0.001) (Table 1). The alfalfa + N treatment increased the soil inorganic P fractions after the rice harvested, and the fractions of NaHCO_3_-Pi, NaOH-Pi and Olsen-P increased in comparison to the control and N fertilizer treatment (Table 2). However, the un-absorbed P fractions, including the NaOH-Po and HCl-P fractions, did not significantly increase from alfalfa additions. The two green manures, with or without N fertilizer application, increased the organic available P content (NaHCO_3_-Po), total labile P fractions and total P content in comparison to the control and N fertilizer treatments, with a higher increase in alfalfa treatment. Compared to the control treatment, the total labile P fractions were increased by 32.52%, 27.68%, 0.20%, 40.98% and 24.04% in alfalfa, broad bean, N fertilizer, alfalfa + N and broad bean + N treatments, respectively (Table 2).

### Soil enzyme activity

The activities of dehydrogenase, urease, acid phosphatase, and β-glucosidase were not significantly different between the N fertilizer and control treatments. Green manure application significantly affected the four enzyme activities (*P* < 0.001) (Table 1). The alfalfa treatment alone significantly increased the activities of dehydrogenase and urease in the two years, and acid phosphatase in 2012, whereas the alfalfa + N treatment significantly increased all the four enzyme activities and had relatively higher enzyme activities of urease, acid phosphatase and β-glucosidase compared to alfalfa treatment alone. The broad bean with or without N fertilizer application increased the β-glucosidase activity (Table 3). The interactive effects between green manure and N fertilizer on the enzyme activities of acid phosphataseand β-glucosidase were significant (*P* < 0.05) (Table 1).

### Correlations among grain yield, soil P fractions and enzyme activities

There were close relationships between grain yield with the fractions of NaHCO_3_-Pi, NaOH-Pi and soil total P, as well as with the enzyme activities of β-glucosidase, acid phosphatase and urease (*P* < 0.05) (Table 4). The fractions of NaHCO_3_-Pi, NaHCO_3_-Po and NaOH-Pi were all significantly correlated with the activities of β-glucosidase, acid phosphate and urease, whereas dehydrogenase activity was significantly correlated with NaHCO_3_-Po and NaOH-Pi fractions (*P* < 0.05). The correlations of urease, acid phosphatase and β-glucosidase were significant in each other, and the correction between dehydrogenase and acid phosphatase was also significant (*P* < 0.05).

The soil P fractions and enzyme activities directly or indirectly affected grain yields. The direct effect coefficients (DECs) of NaHCO_3_-Pi, NaOH-Pi, acid phosphatase and urease on the grain yield were high and positive, but the soil total P and HCl-P had negative direct effects on grain yield. The indirect effect coefficient (IDECs) of the NaHCO_3_-Po via the NaHCO_3_-Pi fraction on grain yield was negatively significant (*P* < 0.05), indicating that the NaHCO_3_-Po fraction indirectly played an important role in increasing the grain yield via the NaHCO_3_-Pi fraction. The IDECs of dehydrogenase via the NaHCO_3_-Po fraction, urease via NaHCO_3_-Pi, β-glucosidase via NaHCO_3_-Pi and NaHCO_3_-Po fractions were also significant and positively affected the grain yield, whereas the IDECs of acid phosphate and urease via the HCl-P fraction were negative, and the increased activities of acid phosphate and urease would reduce the HCl-P fraction and increase the grain yield as a result (Table 5).

## Discussion

### Alfalfa green manure increased rice yield by enhancing P accumulations in rice

Plant growth is strongly associated with its ability to absorb certain nutrients efficiently, and expresses a significant positive correlation between plant growth and foliar concentrations of N and P^33^. In the present study, the N amount input into soil with alfalfa green manure application was 34.30% lower than that in N fertilizer treatment, and leaf N concentration of rice was also greatly lower in alfalfa green manure treatment than the N fertilizer treatment. Based on the results, it was thought that the grain yield in alfalfa treatment should be greatly lower than N fertilizer treatment, but in fact there were no significant differences in grain yields between the two treatments. The mechanism of the positive effect of alfalfa green manure on increasing grain yield under low N nutrient level has not been clearly elaborated. A quantitative relationship between leaf N and P concentrations determine the association that exists between N and P in protein building^34^, and thus an adequate N and P accumulation in leaves is essential for sustaining plant growth and maximizing crop yield^35^,^36^. In the present study, P concentrations and accumulations in leaves, straw, and grain in alfalfa treatment were all higher than that in the N fertilizer treatment. The high leaf P concentration had significant direct effect and indirect effect on increasing rice grain yield, and the indirect effect was via the leaf N concentration^32^. This great interaction between N and P in the rice leaves of alfalfa treatment promoted the rice growth and grain yield increase in a certain extent. However, the N amount supplied by alfalfa green manure was not enough to satisfy the demand for the P to maximize grain yield, consequently limited the effect of alfalfa green manure on increasing grain yield. The N fertilizer application combined with alfalfa green manure eliminated the influence of N deficiency and promoted the P efficiency, as a result, the grain yield and aboveground biomass were significantly increased, which was significant higher than either alfalfa or N fertilizer treatment alone. Therefore, promoting P uptake and enhancing interactive effect of N and P would be a major contribution of alfalfa green manure toward increasing grain yields.

### Alfalfa green manure increased the soil available P fractions

Phosphorus exists in soil in various forms including labile and stable forms, and the ratio of different forms of P in soil can influence P uptake and use efficiency for plant. The NaHCO_3_-Pi fraction (including Olsen-P) is the most labile P pool, which is directly absorbed by plants^21^. The NaOH-Pi is associated with Fe oxides, Al oxides and humic substances after a short term period of submergence^37^. The NaOH-Pi fraction represents the moderately labile P pool as they are not immediately available to plants, but has the potential to become available P over a medium period through biological and physio-chemical transformations^21^. Singh *et al*.^38^ reported that a greater amount of labile P was present in green manure application compared to N fertilizer application in a rice-wheat rotation system. In the present study, the labile P fractions (NaHCO_3_-Pi and NaOH-Pi) were significantly increased in the presence of green manures compared to the control and N fertilizer application. The relationships between NaHCO_3_-Pi and NaOH-Pi with grain yield were positively significant, and the NaHCO_3_-Pi and NaOH-Pi contributed a significant direct effect (DEC) on grain yields (Table 5), indicating that green manure application increased grain yields by maintaining a larger amount of soil labile P, in which the alfalfa green manure had more pronounced effect on maintaining soil labile P than broad bean.

The increase in the soil Olsen-P, NaHCO_3_-Pi, and NaOH-Pi fractions after green manure application may be partly due to the following: (1) an additional supply of P through green manure materials is mostly in the available form of orthophosphate^24^; (2) organic acids in green manure decrease the soil P sorption strength on mineral soil particles by competing organic molecules with orthophosphate for P retention sites (Al and Fe oxide blinding sites) and reducing the numbers of binding sites by chelation and solubilization of Al and Fe oxides with organic acids^26^,^27^; (3) low-molecular-weight compounds in green manures and root exudation have similar effects on blocking P sorption sites, but the effects have been found to be only transient^39^; and (4) green manures enhance soil enzyme activities that are responsible for solubilization P^40^. Based on the P input and uptake under the two green manure treatments in the present study, the P accumulation in aboveground biomass of rice was 5.9 kg ha^−1^ higher in alfalfa treatment than broad bean treatment, while the P input into soil was 4.2 kg ha^−1^ higher in the alfalfa treatment than broad bean treatment, and the difference between P input and uptake indicates that the positive effect of alfalfa green manure on increasing soil available P is greater than broad bean, which may be partly attributed to higher orthophosphate input and soil enzyme activities in alfalfa treatment than broad bean treatment.

The soil labile organic P (NaHCO_3_-Po) is easily mineralized, and contributes to plant available P, especially in low P input systems. The immobilization-mineralization of P is strongly controlled by the supply of, and plant need for P^8^. Similar to the NaHCO_3_-Pi fraction, a most significant increase in NaHCO_3_-Po fraction was observed in the alfalfa treatment in this study. Although the direct effect (DEC) of NaHCO_3_-Po on grain yield was not significant, the indirect effect (IDEC) of the NaHCO_3_-Po fraction via the NaHCO_3_-Pi fraction on grain yield was negatively significant. Alfalfa green manure application greatly increased the grain yield by maintaining a larger amount of the soil NaHCO_3_-Po pool, and the NaHCO_3_-Po pool would be transferred to the NaHCO_3_-Pi pool to promote rice growth when the soil labile Pi was deficient.

The HCl-P pool represents non-labile or stable P compounds, such as Ca-bound P and stable humus bound P. Organic P fractions of the moderately labile P pool are represented by the NaOH-Po fraction, which is difficult to mineralize to plant available P. The presence of HCl-P and NaOH-Po fractions are responsible for the long term release of P through pedological processes and are generally assumed to be of low availability to plants. In the present study, the NaOH-Po and HCl-P fractions were not significantly changed by green manure applications in comparison with the control and N fertilizer treatments, and the most of P in the two green manures was transferred to labile P. Net P mineralization is usually positively correlated with organic matter concentrations^41^ and negatively correlated with the C/P ratio^1^. Alfalfa had a higher P concentration and lower C/P ratio (P: 3 g kg^−1^; C/P: 135) compared to broad bean (P: 1.6 g kg^−1^; C/P: 272), and alfalfa green manure applications input a larger amount of P (9 kg ha^−1^ in alfalfa) into rice paddy fields compared to broad bean (4.8 kg ha^−1^), but the NaOH-Po and HCl-P fractions were not significantly different between the two green manure treatments, even the HCl-P fraction was lower in the alfalfa + N treatment than the broad bean + N treatment, indicating that alfalfa green manure has a greater ability for mineralizing organic P and decreasing soil P sorption than broad bean.

### Alfalfa green manure stimulated four enzyme activities in soil

Soil enzyme production is an essential component of assessing biological changes, soil microbial activities and substrate mineralization^42^. Organic amendment can increase some soil enzyme activities, such as phosphatase, dehydrogenase^43^,^44^. This may be a consequence of the stimulation of microbial growth and activity from improving nutrient availability, soil physical properties, and microbial community composition induced by organic matter^45^,^46^,^47^. The changes of soil enzyme activities directly or indirectly affect the progress of nutrients (N and P) mineralization and release. In this study, the four enzyme activities (dehydrogenase, urease, acid phosphatase and β-glucosidase) were increased by green manure additions compared with control. Because alfalfa had higher N and P contents and a lower C/P ratio, which caused higher N and P input and accumulation in soil compared to broad bean, therefore, alfalfa additions with N fertilizer application exhibited a larger effect on promoting enzyme activities than broad bean + N additions. Dehydrogenase is present in all microorganisms^48^ and is often used as an indicator of microbial oxidative activity and total viable microorganisms in soil^42^. The alfalfa green manure significantly increased dehydrogenase activity, which had a significant positive indirect effect (IDEC) on grain yield via NaHCO_3_-Po. Sustaining the larger viable microorganisms requires a high amount of N nutrient, therefore, the activities of urease, acid phosphatase and β-glucosidase reached to the highest level after N fertilizer application in the alfalfa treatment, correspondingly P accumulations in aboveground biomass and soil available P pool also reached to the highest level among all treatments. The decreased dehydrogenase and β-glucosidase activities in the N fertilizer treatment could be partially attributed to the long-term overuse of chemical fertilizers in this paddy field, deteriorated biological properties and biochemical functions of the soil. Thus, alfalfa green manure application combined with N fertilizer may produce a favourable environment for soil microorganism, and increase the total viable microorganisms which accelerate the mineralization of organic P in green manure and soil.

Soil phosphatase is responsible for soil organic P mineralization into inorganic form and thus, it plays a critical role in regulating the P cycle for crop nutrition, especially in P deficient soils^49^. Urease and β-glucosidase are two key enzymes in regulating organic N hydrolyzation to ammonium^50^, and release energy sources for microorganisms^51^. Thus, the three enzymes are charged with different functions in the process of organic matter decomposition. In the present study, the three enzyme activities were closely related to the fractions of labile Pi and NaHCO_3_-Po. The increased activities of acid phosphatase and urease in green manure application, especially in alfalfa application, directly increased the fractions of labile Pi and NaHCO_3_-Po, and resulted in a significant direct effect (DEC) on grain yields. Although the direct effect of β-glucosidase on grain yields was not significant, β-glucosidase indirectly affected grain yields by influencing the NaHCO_3_-Pi and NaHCO_3_-Po fractions. Thus, the alfalfa green manure induced high total soil available P is closely related to the increased activities of dehydrogenase, urease, acid phosphatase and β-glucosidase.

## Conclusions

Labile P deficiency is a major factor limiting rice growth in paddy soils. Alfalfa green manure, with higher N and P concentrations, as well as lower C/N and C/P ratios, significantly increased the soil available P through a high amount of organic P input and transformation into labile P. This increase of soil available P was closely related to alfalfa addition induced high soil enzyme activities, including dehydrogenase, urease, acid phosphatase and β-glucosidase, which played significantly direct and indirect effects on grain production. The positive effect of alfalfa green manure on increasing grain yield under low N nutrient level can be attributed to its good functions on increasing soil available P, promoting P uptake and enhancing interactive effect of N and P. This distinct ability of alfalfa green manure is very useful to reduce chemical fertilizer application in agroecosystem, maintain a sustainable P supply for rice cultivation, and reduce river eutrophication.

## Materials and Methods

### Site description and experimental design

The field trial was performed in a paddy field at Minhang county, Shanghai, China (located at 121.47 °E, 31.13 °N) in 2012 and 2014. The climate belongs to northern subtropical monsoon climate with average annual temperature of 15.3 °C, annual frost-free period of 225 d and annual rainfall of 1022 mm. The experimental field had been used to plant rice for several decades, and the soil is a viscidity, with pH (1:5 water) 5.24. The soil nitrogen, Olsen-P, available potassium, and organic carbon in the depth of 0–10 cm are 1.03 g kg^−1^, 17.47 mg kg^−1^, 105.14 mg kg^−1^, and 11.22 g kg^−1^, respectively, on May 2012.

The experiment used a split-split plot design: two green manure species (alfalfa and broad bean), and two N fertilization levels (0 and 200 kg ha^−1^), alone or combination, were applied to rice paddy soils in 2012 and 2014. The broad bean was a local and traditional cultivated green manure crop, we selected it as a green manure control to compare it with alfalfa green manure. The treatments were as follows: (1) no application of N fertilizer and green manure (control treatment); (2) alfalfa green manure application (3000 kg dry matter (DM) ha^−1^, alfalfa treatment); (3) broad bean green manure application (3000 kg DM ha^−1^, broad bean treatment); (4) nitrogen fertilizer application (200 kg N ha^−1^, N fertilizer), which was in accordance with local N application amount in our experimental site; (5) a combined treatment of alfalfa (3000 kg DM ha^−1^) and N fertilizer (200 kg N ha^−1^) (alfalfa + N); and (6) a combined treatment of broad bean (3000 kg DM ha^−1^) and N fertilizer (200 kg N ha^−1^) (broad bean + N). The treatments had three replicates, randomly distributed in blocks with plot sizes of 10 m^2^ (2 m × 5 m).

### Green manure and crop management

Green manure and fertilizer treatments: Ten days prior to transplanting rice, alfalfa and broad bean were harvested from other place, transported to experimental site, chopped into 5–8 cm pieces, and incorporated manually to a depth of 15 cm. Calcium superphosphate (P_2_O_5_ content 20%) was incorporated into soil as base fertilizer at net P rates of 20 kg ha^−1^ in the same time. All plots were then flood irrigated to a depth of approximately 10 cm. The green manure addition was in the form of fresh alfalfa and broad bean. N fertilizer (urea, nitrogen content 46%) was equally divided into two portions (100 kg N fertilizer ha^−1^ per portion) in each year. The divided N fertilizer was applied in rice paddy field on 7^th^ and 30^th^ after rice transplanting.

Rice transplanting: The rice cultivar used in this experiment was Qiuyoujinfeng, which came from the Academy of Agricultural Sciences in Shanghai. Rice seedlings that were 21 days old were transplanted by hand into plots. Plant spacing was 15 cm between each hill in a row (three plants per hill) and 30 cm between each row. Transplanting occurred on June 8, 2012 and June 8, 2014, and plants were harvested on November 10 in 2012 and October 30 in 2014.

Water management in our experiment followed local practices of rice cultivation. Water levels were maintained at 5–8 cm above the ground for all treatments before the mature stage of the rice, except for one week dry stage to control the number of rice tillers at the end of the tillering stage.

The nutrient quality of green manures: the biomass yields of alfalfa and broad beans in dry weight were 15 Mg ha^−1^ year^−1^ 52 and 9 Mg ha^−1^ year^−1^ according to the local traditional experience. In order to easily compare the two green manures, we used the same amount of alfalfa and broad beans (3000 kg ha^−1^) in dry weight as green manure in our study. The average C, N, and P concentrations were, respectively, 40.50%, 4.38% and 0.30% in alfalfa and 43.44%, 2.49%, and 0.16% in broad beans. The average amounts of C, N, and P in the 3000 kg ha^−1^ green manure were, respectively, 1215 kg, 131.4 kg, and 9.0 kg in alfalfa and 1303.2 kg, 74.7 kg, and 4.8 kg in broad beans.

### Soil and rice sampling

Soil samples were collected (0–10 cm) on the rice harvest dates with a 5 cm diameter auger, and three samples were taken along the diagonal in each plot and mixed together as a replicate. After sampling, the soil samples were divided into two portions, one portion was sieved (<4 mm), mixed and frozen immediately (−20 °C), and was used for analyzing enzyme activity within a month. Another portion was air-dried and sieved (<2 mm) for assaying the soil P. The concentration of P and enzyme activities of soil were calculated on the basis of oven-dry (105 °C) weight.

Leaf samples: First leaf on top of rice plant was collected at three growth stages, which were on the 30^th^ (tillering stage), 80^th^ (panicle stage) and 110^th^ (filling stage). More than 20 leaves were collected from different plants randomly in each plot. The leaves of the three stages was mixed together in equal weight in each plot, and then cushed and sieved (<2 mm) to analyze N and P concentrations.

Rice samples, with areas of 1 m × 1 m, were harvested three times in each plot for recording the grain and straw yields. The average of the three cuttings was as a replicate in each plot. Leaf and straw were first dried at 105 °C for half an hour, then dried at 65 °C until a constant weight. The grain was dried at 65 °C to a constant weight.

### Chemical analysis

Nitrogen (N) concentrations of leaves, straw and grain were determined by elemental analyzer (Vario EL III, Elementar, Germany).

The soil samples for Olsen-P analysis were extracted with sodium bicarbonate (NaHCO_3_) (0.5 M, pH 8.5, 1:20; soil: extract ratio, 30 min shaking); the leaf, grain, straw and soil samples were digested with sulfuric acid (H_2_SO_4_)/ perchloric acid (HClO_4_) (10:1) to determine the P concentration. The P concentration was analyzed used an atomic absorption spectrophotometer (AA—6800 F, Shimadzu Corporation, Japan).

N and P accumulations in grain and straw were calculated by multiplying N or P concentrations with yields of grain or straw per unit area, respectively. N and P accumulations in the total aboveground biomass include N and P accumulations in grain and straw.

Soil P fractionation: the soil samples were subjected to P fractionation by using a modified version of the Hedley^53^, as described by Zhang and Mckenzie^54^. Processed soil samples (0.5 g samples passed through a 2 mm sieve) in duplicate were sequentially extracted with 30 ml each of 0.5 M NaHCO_3_ (pH 8.5), 0.1 M NaOH, and 1.0 M HCl by shaking the samples for 16 h. After shaking, the suspensions were centrifuged for 10 min at 8000 rpm and then filtered through 0.45 μm filters (Whatman International Ltd., China). The soil residue was digested with H_2_SO_4_/HClO_4_ to determine the residual P. The inorganic P (Pi) concentration in a previously neutralized (using 0.9 M H_2_SO_4_) extract was determined using an atomic absorption spectrophotometer (AA—6800 F, Shimadzu Corporation, Japan). A portion of aliquot obtained by extraction with NaHCO_3_ and NaOH was digested with acidified ammonium-per-sulfate ((NH_4_)_2_S_2_O_8_) and analyzed for the total P. The concentration of organic P (Po) was calculated as the difference between the total P and inorganic P (Pi). The total P in HCl extracts was also digested with acidified ammonium-per-sulfate, and the P in the HCl extracts is commonly the inorganic P (Pi).

Soil enzyme activity: the acid phosphatase (E.C. 3.1.3.2) and β-glucosidase (3.2.1.21) assay was based on *p*-nitrophenol (NPP) release after the cleavage of a synthetic substrate *p*-nitrophenyl phosphate (*p*NPP) (Sigma, UK)^42^. For the acid phosphatase assay, 1 g soil (wet weight) was mixed with 4 ml of 500 mM modified universal buffer (pH 4.0) and a 1 ml substrate (15 mM). The control contained 4 ml of modified universal buffer and 1 ml of sterile distilled water. The soils were vortexed briefly and then incubated (20 °C, 200 rev min^−1^) on an orbital shaker for 2 h. Next, 1 ml of sterile distilled water was added to the samples and 1 ml of substrate was added to the controls before the reaction was terminated with the addition of 1 ml of 500 mM CaCl_2_ and 4 ml of 500 mM NaOH. The suspensions were shaken on an orbital shaker (20 °C, 200 rev min^−1^) for 30 min. The aliquots (1.5 ml) were centrifuged (9464 × *g*, 5 min) and the color intensity of the extracted *p*-nitrophenol was measured at 400 nm (Hellos Gamma, England). A standard curve was plotted using a range of *p*-nitrophenol (Sigma, UK) concentrations between 0 and 50 μg ml^−1^ of distilled water.

The β-glucosidase assays differed from the above only in the choice of buffer (modified universal buffer pH 6.0), and the substrate concentration was 25 mM. The INT (2(*p-*iodophenyl)-3-(*p*-nitrophenyl)-5-phenyl tetrazo-lium chloride) reductase activity (i.e., dehydrogenase activity) was determined according to Von Mersi and Schinner^55^. Briefly, 1 g (wet weight) soil was placed in foil-wrapped universal bottles and mixed with 1.5 ml of 1 M Tris buffer of pH 7.0 and 2 ml INT (5 mg ml^−1^ in 2% v/v *N, N*-dimethylformamide). The control soils received a 1.5 ml Tris buffer and 2 ml distilled water. The samples were incubated in an orbital shaker (20 °C, 200 rev min^−1^) for 24 h. Then, 2 ml of distilled water were added to the sample soils and 2 ml INT were added to the control soils. The reaction was stopped by adding 10 ml *N, N*-dimethylformamide/ethanol (1:1 ratio) extractant and by shaking (20 °C, 200 rev min^−1^) for 1 h. The aliquots (1.5 ml) were removed and centrifuged (9464 × *g*, 5 min) and the absorbance of the supernatants was measured at 464 nm. The Tris buffer was replaced with distilled water for the natural soil pH assay. A standard curve was obtained using INTF (iodonitrotetrazolium chioride) (Sigma, UK) at a concentration of 0–27 μg ml^−1^ extractant.

A modified assay for urease (EC 3.5.1.5) activity, based on that of Kandeler and Gerber^56^ was used. The soil (5 g wet weight) was mixed with 2.5 ml urea (80 mM) and 20 ml of 75 mM borate buffer (pH 10.0). The mixture was reacted for 4 h in an orbital shaker (20 °C, 200 rev min^−1^). The controls were prepared by addition of 2.5 ml of sterile distilled water and 20 ml borate buffer. After 4 h, 2.5 ml of sterile distilled water was added to the treatment and 2.5 ml urea was added to the controls before extraction with a 30 ml acidified 2 M KCl. The suspensions were shaken (20 °C, 200 rev min^−1^) for 30 min. The aliquots (1.5 ml) were centrifuged (9464 × *g*, 5 min) and 1 ml of the supernatant fraction was mixed with 9 ml of distilled water, 5 ml sodium salicylate/NaOH solution and 2 ml dichloroisocyanuric acid (Na^+^ salt). The color intensity of the solution, after standing 20 ± 2 °C for 1 h, was measured at 690 nm. The borate buffer was replaced by distilled water for the natural soil pH assay. The ammonium concentrations were determined using a calibration curve of ammonium chloride standard solutions from 0 to 2.5 μg ml^−1^.

### Statistical analysis

The treatment differences were tested using a mean separation test that named the least significant difference (LSD) at a *P* = 0.05 level. The effects of N fertilization and green manures on grain yield, plant and soil properties were examined by using a two-way ANOVA analysis. The correlation coefficients were calculated using Pearson’s method at *P* = 0.05 (SAS 9.0 Institute Inc., Cary, NC, USA). The direct effect coefficients (DECs) were determined using SAS 9.0, and the indirect effect coefficients (IDECs) were calculated using SAS 9.0 based on the Pearson correlation coefficients.

## Additional Information

**How to cite this article**: Gao, X. *et al*. Increase phosphorus availability from the use of alfalfa (*Medicago sativa* L) green manure in rice (*Oryza sativa* L.) agroecosystem. *Sci. Rep.* **6**, 36981; doi: 10.1038/srep36981 (2016).

**Publisher’s note:** Springer Nature remains neutral with regard to jurisdictional claims in published maps and institutional affiliations.

## Figures and Tables

**Table 1:** Effects of N fertilization and green manure on grain yield, nutrient accumulation, soil available P, and soil enzyme activities in 2012 and 2014.

Treatment	Grain yield (Mg ha^−1^)	Leaf N concentration (g kg^−1^)	N accumulation in total above ground biomass (kg ha^−1^)	Leaf P concentration (g kg^−1^)	Grain P concentration (g kg^−1^)	P accumulation in total above ground biomass^a^ (kg ha^−1^)	Soil available P^b^ (mg kg^−1^)	Dehydrogenase INTF mg kg^−1^24h^−1^)	Urease (NH_4_^+^-N mg kg^−1^4h^−1^)	Acid phosphatase (*p*-nitrophenol mg kg^−1^2h^−1^)	β-glucosidase (*p*-nitrophenol mg kg^−1^ 2 h^−1^)
N fertilizer (F)											
0	8.93^b^	22.21^b^	155.98^b^	1.96^a^	1.86^a^	25.46^b^	192.40^a^	11.72^a^	225.10^b^	438.62^b^	234.58^a^
N	10.45^a^	29.75a	249.21a	2.07^a^	1.82^a^	28.39^a^	194.76^a^	10.97^b^	245.23^a^	448.63^a^	232.34^a^
Green manure (G)											
None	8.97^c^	23.73^c^	162.79^c^	1.70^c^	1.62^c^	20.86^c^	160.32^c^	10.28^c^	213.46^c^	422.95^b^	197.85^b^
alfalfa	10.40^a^	28.29^a^	237.22^a^	2.36^a^	2.00^a^	32.41^a^	218.98^a^	12.65^a^	255.53^a^	458.16^a^	251.48^a^
broad bean	9.70^b^	25.93^b^	207.77^b^	1.99^b^	1.90^b^	27.46^b^	201.44^b^	11.12^b^	236.52^b^	449.76^a^	251.04^a^
F	***	***	***	NS	NS	***	NS	*	**	*	NS
G	***	***	***	***	***	***	***	***	***	***	***
F*G	*	*	*	NS	**	***	NS	NS	NS	*	*

^a^Total above ground biomass including yields of grain and straw;

^b^Soil available P including soil NaHCO_3_-Pi, NaOH-Pi, and NaHCO_3_-Po;

Within each main treatment of F and G, means followed by different letters in the same column are significantly different at *P* < 0.05. *Significant at *P* < 0.05; **Significant at *P* < 0.01; ***Significant at *P* < 0.001; NS, not significant.

**Table 2:** Different fractionations of P as affected by N fertilizer and green manures (mg kg^−1^).

	NaHCO_3_-Pi	NaHCO_3_-Po	NaOH-Pi	Total labile P	NaOH-Po	HCl-P	Residual-P	Total-P	Olsen-P
2012									
Control	40.63 ± 0.44^c^	42.32 ± 2.82^b^	72.06 ± 0.65^c^	155.01 ± 3.56^c^	36.42 ± 1.92a,^b^	193.60 ± 1.18^b^^,c^	47.37 ± 3.41^b^	471.98 ± 3.82^c^	18.71 ± 0.21^c^
Alfalfa	46.33 ± 4.25^b^	68.13 ± 4.85^a^	89.43 ± 6.89a,^b^	203.89 ± 7.46a,^b^	36.79 ± 1.22a,^b^	194.94 ± 3.53^b^^,c^	60.84 ± 3.36^a^	536.44 ± 14.26^b^	21.51 ± 0.30^b^
Broad bean	45.54 ± 0.50^b^	63.43 ± 4.49^a^	85.32 ± 5.41^b^	194.29 ± 2.91a,^b^	38.15 ± 1.79^a^	192.18 ± 1.23^c^	50.76 ± 2.21a,^b^	522.40 ± 7.10^b^	20.89 ± 1.38^b^^,c^
N fertilizer	42.80 ± 2.58^c^	30.82 ± 2.74^b^	84.06 ± 3.60^b^	157.68 ± 3.93^c^	35.57 ± 0.54a,^b^	196.99 ± 0.13^b^^,c^	44.43 ± 3.12^b^^,c^	494.73 ± 8.07^b^^,c^	19.63 ± 1.08^b^^,c^
Alfalfa + N	58.32 ± 1.84^a^	63.79 ± 5.02^a^	103.16 ± 8.81^a^	225.27 ± 15.56^a^	34.16 ± 2.65a,^b^	199.54 ± 2.67a,^b^	47.67 ± 5.21^b^	601.08 ± 11.10^a^	24.67 ± 0.83^a^
Broad bean + N	48.64 ± 0.51^b^	62.81 ± 4.51^a^	95.55 ± 1.64a,^b^	207.00 ± 6.66a,^b^	30.41 ± 3.43^b^	204.72 ± 0.96^a^	35.27 ± 2.66^c^	537.33 ± 29.71^b^	22.05 ± 0.29^b^
2014									
Control	42.76 ± 1.11^c^	43.35 ± 0.54^b^	79.28 ± 2.70^c^	165.39 ± 3.81^c^	37.26 ± 0.85^a^	182.58 ± 4.00^b^	46.65 ± 0.72^b^^,c^	442.15 ± 8.11^d^	16.16 ± 0.38^b^^,c^
Alfalfa	48.21 ± 1.61^b^	72.02 ± 0.57^a^	100.56 ± 4.37^a^	220.79 ± 5.69^a^	36.70 ± 2.68^a^	270.55 ± 17.18^a^	59.21 ± 3.04^a^	652.54 ± 22.85^a^	19.20 ± 0.53^a^
Broad bean	47.80 ± 0.89^b^	63.14 ± 6.19^a^	104.11 ± 1.08^a^	215.05 ± 4.82^a^	32.23 ± 2.29a,^b^	265.70 ± 16.71^a^	54.59 ± 3.45a,^b^	627.56 ± 24.61a,^b^	18.53 ± 1.456^a^
N fertilizer	33.97 ± 2.63^d^	44.00 ± 3.76^b^	85.24 ± 1.58^b^^,c^	163.21 ± 1.59^c^	26.59 ± 1.74^b^	210.92 ± 21.84^a^	47.20 ± 2.48^c^	476.27 ± 18.32^d^	14.22 ± 0.49^c^
Alfalfa + N	47.38 ± 0.88^b^	70.97 ± 5.56^a^	107.62 ± 3.40^a^	225.97 ± 4.79^a^	34.06 ± 2.08^a^	191.86 ± 9.43^b^	54.70 ± 0.51a,^b^	595.77 ± 13.54^b,^^c^	17.98 ± 0.65a,^b^
Broad bean + N	54.23 ± 1.46^a^	45.80 ± 4.53^b^	89.40 ± 4.29^b^	189.43 ± 9.32^b^	31.57 ± 0.92a,^b^	217.23 ± 7.62^a^	40.76 ± 0.17^c^	554.19 ± 17.69^c^	17.70 ± 0.46a,^b^

Data are means ± standard errors.

^a^Pi: inorganic P, Po: organic P.

^b^The total labile P including NaHCO_3_-Pi, NaHCO_3_-Po, and NaOH-Pi fractions.

The different letters in the same column in the same year are significant (*P* < 0.05).

**Table 3:** Soil enzymes as affected by N fertilizer and green manures. Data are means  ±  standard errors.

	Dehydrogenase (INTF mg kg^−1^ 24 h^−1^)	Urease (NH_4_^+^-N mg kg^−1^ 4 h^−1^)	Acid phosphatase (*p*-nitrophenol mg kg^−1^ 2 h^−1^)	β-glucosidase (*p*-nitrophenol mg kg^−1^ 2 h^−1^)
2012				
Control	9.61 ± 0.42 ^cd^	216.73 ± 9.58^b^	396.75 ± 1.48^d^	207.62 ± 8.33^b^
Alfalfa	13.30 ± 0.30^a^	258.72 ± 4.81^ab^	451.24 ± 14.08^abc^	254.83 ± 9.95^a^
Broad bean	10.86 ± 0.43^bc^	237.64 ± 23.28^b^	463.5 ± 5.58^ab^	251.30 ± 18.19^a^
N fertilizer	9.02 ± 0.50^d^	230.05 ± 11.25^b^	436.25 ± 4.51^c^	193.23 ± 8.79^b^
Alfalfa + N	11.23 ± 0.88^b^	296.98 ± 17.42^a^	470.26 ± 1.76^a^	269.00 ± 14.48^a^
Broad bean + N	10.23 ± 0.38^bcd^	248.72 ± 7.14^b^	445.52 ± 5.51^bc^	247.47 ± 13.70^a^
2014				
Control	11.50 ± 0.68^bc^	193.95 ± 0.90^d^	428.35 ± 5.49^c^	208.18 ± 12.14^bc^
Alfalfa	13.25 ± 0.83^a^	223.57 ± 9.63^abc^	451.08 ± 9.48^a^	220.96 ± 15.73^b^
Broad bean	11.82 ± 0.50^abc^	220.01 ± 6.73^bc^	440.77 ± 8.86^abc^	264.56 ± 8.73^a^
N fertilizer	10.97 ± 0.46^c^	213.09 ± 3.14 ^cd^	430.43 ± 5.18^bc^	182.38 ± 13.44^c^
Alfalfa + N	12.82 ± 0.21^ab^	242.85 ± 8.32^a^	460.05 ± 3.83^a^	261.14 ± 6.51^a^
Broad bean + N	11.56 ± 0.46^abc^	239.71 ± 8.91^ab^	449.26 ± 1.57^ab^	240.82 ± 12.27^ab^

The different letters in the same column in the same year are significant (*P* < 0.05).

**Table 4:** Correlation coefficients between grain yield and soil P fractions, enzyme activities across 2012 and 2014.

Factors	Grain yield	NaHCO_3_-Pi^a^	NaHCO_3_-Po^a^	NaOH-Pi	NaOH-Po	HCl-P	Residue-P	Total-P	Dehydrogenase	β-glucosidase	Acid phosphatase	Urease
Grain yield	1	0.51**	0.26	0.49**	−0.17	−0.06	−0.09	0.38*	0.25	0.34*	0.56***	0.54***
NaHCO_3_-Pi		1	0.36*	0.58***	0.13	0.17	−0.09	0.60***	0.21	0.64***	0.43**	0.59***
NaHCO_3_-Po			1	0.59***	0.05	0.27	0.43**	0.65***	0.59***	0.60***	0.56***	0.33
NaOH-Pi				1	−0.08	0.43**	0.26	0.73***	0.39*	0.54***	0.46**	0.35*
NaOH-Po					1	−0.1	0.27	−0.06	0.11	0.07	0.02	−0.02
HCl-P						1	0.29	0.64***	0.26	0.11	0.08	−0.16
Residue-P							1	0.32	0.52**	0.11	0.2	−0.1
Total-P								1	0.47**	0.52**	0.53***	0.36*
Dehydrogenase									1	0.24	0.38*	0.08
β-glucosidase										1	0.49**	0.51**
Acid phosphatase											1	0.51**
Urease												1

^a^Pi: inorganic P, Po: organic P.

*Significant *P* < 0.05, ^**^Significant *P* < 0.01, ***Significant *P* < 0.001.

**Table 5:** Direct and indirect effects of soil P fractions and soil enzyme activities on grain yield.

Factors	Direct effect coefficients	Indirect effect coefficients										
		NaHCO_3_-Pi	NaHCO_3_-Po	NaOH-Pi	NaOH-Po	HCl-P	Residue-P	Total-P	Dehydrogenase	β-glucosidase	Acid phosphatase	Urease
NaHCO_3_-Pi	**0.32**		−0.31*	0.33*	−0.02	0.61*	−0.53*	0.29*	0.22	0.46*	−0.08	0.38*
NaHCO_3_-Po	−0.15	−0.27*		0.18	−0.01	0.09	0.10	0.22	0.45*	0.49*	0.16	0.04
NaOH-Pi	**0.27**	0.24	0.15		0.01	−0.28	0.17	0.11	−0.02	0.06	0.03	−0.02
NaOH-Po	−0.16	−0.23	0.03	−0.14		−0.04	0.32*	0.07	−0.02	−0.01	0.01	−0.09
HCl-P	−**0.23**	−0.09	−0.15	0.09	0.02		0.06	0.45*	−0.04	−0.01	−0.23	−0.39*
Residue-P	−0.11	−0.29*	0.06	0.12	−0.04	0.48*		0.08	0.33*	0.05	0.01	−0.01
Total-P	−**0.43**	0.48*	0.41*	0.27	0.01	−0.38*	0.24		0.08	−0.11	0.41*	0.26
Dehydrogenase	0.18	0.12	0.27*	−0.01	−0.02	−0.03	0.32*	0.02		−0.17	0.11	−0.13
β-glucosidase	−0.17	−0.32*	0.38*	0.06	−0.01	−0.02	0.06	−0.04	−0.22		0.07	0.08
Acid phosphatase	**0.41**	−0.05	0.09	0.02	0.00	0.02	0.01	0.13	0.11	0.06		0.23
Urease	**0.35**	0.23	0.02	−0.02	0.00	−0.14	−0.01	0.09	−0.14	0.07	0.26	

*significant *P* < 0.05.
